# Time trends in the burden of stroke and subtypes attributable to PM2.5 in China from 1990 to 2019

**DOI:** 10.3389/fpubh.2022.1026870

**Published:** 2022-10-12

**Authors:** Huijing Chen, Zhihua Zhou, Zhenglong Li, Shanshan Liang, Jingjing Zhou, Guanyang Zou, Shangcheng Zhou

**Affiliations:** School of Public Health and Management, Guangzhou University of Chinese Medicine, Guangzhou, China

**Keywords:** PM2.5, stroke, Joinpoint regression, age-period-cohort model, ambient particulate matter pollution, household air pollution

## Abstract

**Background:**

Increasing studies have found that PM2.5 has large adverse effects on stroke mortality. We want to investigate the long-term trends in the mortality of stroke attributable to ambient particulate matter pollution and household air pollution to provide evidence facilitating the design of policy.

**Methods:**

The deaths data of stroke and its subtypes attributable to PM2.5 were obtained from the Global Burden of Disease (GBD) 2019, analyzed by Joinpoint regression software and the age-period-cohort (APC) method to assess the magnitude of the trends in mortality and the temporal trends in the mortality rate by age, period, and cohort.

**Results:**

From 1990 to 2019, the age-standardized mortality rate (ASMR) attributable to PM2.5 exposure trended downwards, but the trends of ambient particulate matter pollution and household air pollution were opposite. The trends varied among subtypes, the AAPC of intracerebral hemorrhage, ischemic stroke, and subarachnoid hemorrhage attributable to PM2.5 were 0.7, 2.5, and−3.3%, respectively. The longitudinal age curve of the APC model showed that the mortality rates due to PM2.5 exposure increased with age. The period RRs of ischemic stroke due to ambient particulate matter pollution increased significantly. The cohort RRs of ambient particulate matter pollution increased among those born from 1905 to 1990. The net drifts of all subtypes attributable to PM2.5 were below 0, but owing to the increase of ambient particulate matter pollution, the range of the decline was small. Males had higher net drift values, compared with females.

**Conclusions:**

Ambient particulate matter pollution has become the main type of PM2.5 leading to stroke in China. PM2.5 exposure is more harmful to ischemic stroke, males, and elderly. Chinese government should pay attention to the long-term impact of ambient air pollution on stroke and take effective public health policies and interventions.

## Introduction

Stroke is a leading cause of mortality in middle-income countries, especially in China (1). According to GBD 2019, the incident rate of stroke in China reached 276.7 per 100,000 population in 2019, an increase of 86.0% over 1990 (2). Previous studies showed that the disease burden of stroke could be mainly attributable to important environmental and lifestyle risk factors (3, 4).

PM2.5, the particulate matter with an aerodynamic diameter ≤ 2.5 μm, has become one of the three risk factors resulting in more than 1% of DALYs globally (5, 6), causing 8.42 million attributable deaths and the $4.09 trillion of health cost in 2016 (7). PM2.5 exposure includes ambient particulate matter pollution exposure and household air pollution exposure. Ambient particulate matter pollution is defined as annual average daily exposure to outdoor air concentrations of PM2.5, which mainly comes from traffic, factories and household fuel, and household air pollution is defined as individual exposure to PM2.5 due to the use of solid cooking fuel (8). Growing amounts of evidence suggest that ambient particulate matter pollution and household air pollution are closely related to stroke.

A European study showed that for every 5 μg/m^3^ increase in environmental PM2.5 per year, the risk of stroke increased by 19% (9). A prospective cohort study (China-PAR) found that for each increase of 10 μg/m^3^ in ambient PM2.5 concentration, the increased risks of incident stroke, ischemic stroke, and hemorrhagic stroke were 13% (hazard ratio 1.13; 95% confidence interval 1.09 to 1.17), 20% (1.20, 1.15 to 1.25), and 12% (1.12, 1.05 to 1.20), respectively (10). It also has found that household air pollution was related to high blood pressure and escalated mortality of stroke (11, 12).

Owing to economic development and the acceleration of industrialization and urbanization, air pollution has become a global social and environmental issue, especially in developing countries such as China (13–15). Therefore, it is very important to evaluate the stroke burden attributable to PM2.5 exposure in China to advance evidence-informed prevention plans. Several studies are exploring the relationship between stroke burden and PM2.5 exposure (16–19), but few of them focus on estimating the temporal trend and analyzing the independent effects of chronological age, period, and birth cohort of three stroke subtypes burden attributable to ambient particulate matter pollution and household air pollution, respectively.

To address these limitations, we collected data from GBD 2019 to estimate the average annual percent change (AAPC) of stroke and three subcategories of mortality attributable to ambient particulate matter pollution and household air pollution, and analyzed the independent effects by an age-period-cohort (APC) mode. The finding of our study may help public health managers to make evidence-based policies and assess specific interventions.

## Materials and methods

### Data sources

We extracted the mortality rate of stroke, intracerebral hemorrhage, ischemic stroke, and subarachnoid hemorrhage attributable to PM2.5 in China from 1990 to 2019 from the Global Burden of Disease (GBD) 2019 at GBD Data tool.

The original stroke mortality data were obtained from the Cause of Deaths Reporting System of the Chinese Centers for Disease Control and Prevention (CDC) and Disease Surveillance Points (DSPs) (20). Ischemic stroke is an episode of neurological dysfunction caused by focal cerebral, spinal, or retinal infarction. Intracerebral hemorrhage is a focal collection of blood within the brain parenchyma or ventricular system that is not caused by trauma. Subarachnoid hemorrhage is bleeding into the subarachnoid space. Stroke and each category are identified by International Classification of Diseases (ICD) version 10 (ICD-10): stroke (G45-G46.8, I60-I62, I62.9-I64, I64.1, I65-I69.998, Z82.3), ischemic stroke (G45-G46.8, I63-I63.9, I65-I66.9, I67.2-I67.848, I69.3-I69.4), intracerebral hemorrhage (I61-I62, I62.9, I69.0-I69.298) and subarachnoid hemorrhage (I60-I60.9, I67.0-I67.1) (21).

The mortality rates were age-standardized as follows:


$$ASMRs=\frac{\sum \begin{array}{l} Age\;composition\;of\;standard\;group\;population\\ \;\times \;Age\;specific\;mortality \end{array}}{Age\;composition\;of\;the\;standard\;population}$$

Based on published trials or cohort studies, GBD2019 provides a standardized and comprehensive assessment of the magnitude of risk factor exposure, relative risk, and attributable burden of disease by age, sex, and geographies for specific points in a series of time by using spatiotemporal Gaussian process regression, DisMod-MR 2.1, Bayesian meta-regression method and other alternative methods (5, 22).

In the GBD study, PM2.5 included ambient particulate matter pollution and household air pollution. Ambient particulate matter pollution was defined as the population-weighted annual average mass concentration of outdoor PM2.5 exposure, which was obtained from satellite observations of aerosols in the atmosphere, ground measurements, chemical transport model simulations, population estimates, and land-use data. Exposure to household air pollution (HAP) from solid fuels is estimated from both the proportion of individuals using solid cooking fuels and the level of PM2.5 air pollution exposure. Solid fuels in GBD2019 include coal, wood, charcoal, dung, and agricultural residues (2, 5). The data of household air pollution were obtained from Demographic and Health Surveys (DHS), Living Standards Measurement Surveys (LSMS), Multiple Indicator Cluster Surveys (MICS), and World Health Surveys (WHS), as well as country-specific survey series such as China Monitoring Survey and South Asia General Household Survey (5, 8).

Population attributable fraction (PAF) represents the proportion of related risk that would be reduced if the exposure to a risk factor were reduced to the theoretical minimum exposure level, which is known as the theoretical minimum risk exposure level (TMREL). In GBD 2019, the TMREL of ambient and household air pollution was between 2.4 and 5.9 μg /m^3^ (8, 17, 23). The calculation formula for attributable deaths (ADs) is as follows (24): AD = PAF ^*^ The number of deaths due to stroke or the subtypes.

The age-standardized rates of PM2.5-ADs, ambient particulate matter pollution -ADs, and household air pollution-ADs were calculated by the world standard population (25). A comprehensive description of the metrics, data sources, and statistical modeling has been reported in GBD 2019 study (5).

### Joinpoint regression analysis

In our studies, JoinPoint software (Version 4.9.1.0) was used to calculate the average annual percentage change (AAPC), as well as 95% CIs, which can analyze the magnitude and direction of trends of the mortality rate of stroke and different subtypes over 30 years. The JoinPoint software estimated the mortality data by applying the grid search method with 5 Joinpoints and the Monte Carlo permutation test to optimize the model.

Suppose there is a sequence of observations (x_1_, y_1_),..., (x_n_, y_n_), of which, x_1_ ≤ ... ≤ x_n_, the JoinPoint regression model can be written in log-liner form as


$$\begin{array}{l} E\left[y_{i}|x_{i}\right]=e^{\beta_{0}+\beta_{1}x_{i}+{\delta_{1}\left(x_{i}-\Upsilon_{1}\right)}^{+}+\ldots {\delta_{k}\left(x_{i}-\Upsilon_{k}\right)}^{+}} \end{array}$$

where y_i_ represents dependent variable and x_i_ denotes independent variable for i = 1, 2, …n; β_0_ represents constant parameter; β_1_ represents regression coefficient; δ_*k*_ represents the regression coefficient of the *kth* piecewise function. When (*x*_*i*_−Υ_*k*_) is over 1, ${\left(x_{i}-\Upsilon_{k}\right)}^{+}=\left(x_{i}-\Upsilon_{k}\right)$, otherwise ${\left(x_{i}-\Upsilon_{k}\right)}^{+}=\;0$ (26).

### Age-period-cohort analysis

It is known that there is collinearity between age, period, and cohort (27). Like previous studies (28, 29), we circumvented this problem by using age-period-cohort (APC) model, which provides a useful parametric framework with complements standard non-parametric descriptive methods, to assess the temporal trends of mortality by age, period, and cohort (30).

The age effect is the age-related physiological and pathological changes, causing epidemiological differences. To assess the age effect, the longitudinal age curve fitted longitudinal age-specific rates, which is relative to the reference cohort adjusted for period deviations. The period effect refers to changes in mortality rate due to human factors, such as the development of diagnosis technology, screening, and early detection, changes in disease definition and registration, treatment improvement, etc. These human factors may affect the disease rate in different periods, resulting in a period effect. The period effect, shown in the period ate ratios (period RRs), refers to changes in disease mortality caused by different human factors, such as the development of disease diagnosis technology, screening, and early detection, changes in disease definition and registration, treatment improvement and the conservation and emission reduction policies introduced by the Chinese government. When RR is over 1, the relative risk of death is higher in this period compared with the reference period, and when RR is below 1,the relative risk of death in this period is lower than that in the reference period. The cohort effects, shown in the cohort rate ratios (cohort RRs), refer to differences in disease mortality rates caused by various lifestyle changes or different exposure to risk factors among generations. Net drift is an overall log-linear trend by calendar period and cohort effects, indicating overall the annual percentage change. Local drift is the log-linear by period and birth cohort for each age group, representing annual percentage changes for each age group (31). Like previous studies (31–33), we also recoded age and period into consecutive 5 years of data. The mortality and population data are arranged into 5-year age groups from 25–29 age-group to 80–84 age-group and 85 plus age-group. Consecutive 5-year periods were defined from 1990–1994 to 2015-2019. Consecutive 5-year cohorts were defined from 1905–1909 to 1985–1989.

APC model can be written in linear regression form as follow (34–36):


(1) $$\begin{array}{l} \text{M}=\mu +\alpha_{i*}\text{age}+\beta_{j*}\text{period}+\gamma_{k*}\text{cohort}+\varepsilon \end{array}$$

M represents the death rate for age *i* age group during *j* period; α_*i*_ denotes age effect of the *i* age group; β_*j*_ represents period effect of the *j* period; γ_*k*_ denotes cohort effect of the k (k = I + j - 1) birth cohort; μ is intercept or adjusted mean death rate, and ε is the residual or a random error.

This part of the statistical analysis was performed by R statistical software (R version 3.5.1) and the age-period-cohort web tool (https://analysistools.cancer.gov/apc/). *p* < 0.05 was considered significant (17, 37).

## Results

### The trend in the age-standardized mortality rate of stroke attributable to PM2.5 exposure

Over the past 30 years, the number of deaths increased 1.3 times over, from 637,871 in 1990 to 801,549 in 2019. The ASMR of stroke attributable to PM2.5 decreased among different stroke subtypes, especially for subarachnoid hemorrhage (decreased 6.7% annually).

We further analyzed the ASMRs of stroke attributable to ambient particulate matter pollution and household air pollution. For ambient particulate matter pollution, the number of deaths increased 3.3 times over, from 161,939 in 1990 to 541,796 in 2019. The ASMR of stroke attributable to ambient particulate matter pollution increased by 1% annually, with similar trends observed among both sexes (AAPC: 1.0%; 95% CI: 0.6, 1.5%). Among different stroke subtypes, the ASMR of subarachnoid hemorrhage decreased (AAPC:−3.3%; 95% CI:−3.7,−2.8%), but the ASMR of ischemic stroke (AAPC: 2.5%; 95% CI: 2.0, 2.9%) and intracerebral hemorrhage (AAPC: 0.5%; 95% CI: 0.3, 0.7%) similarly increased from 1990 to 2019.

For household air pollution, the number of deaths decreased from 382, 505 in 1990 to 136,950 in 2019. It also decreased significantly among both sexes (AAPC: −6.6%; 95% CI: – 6.9, −6.3%). The ASMRs of different stroke subtypes attributable to household air pollution were all decreased.

**Figure 1:**
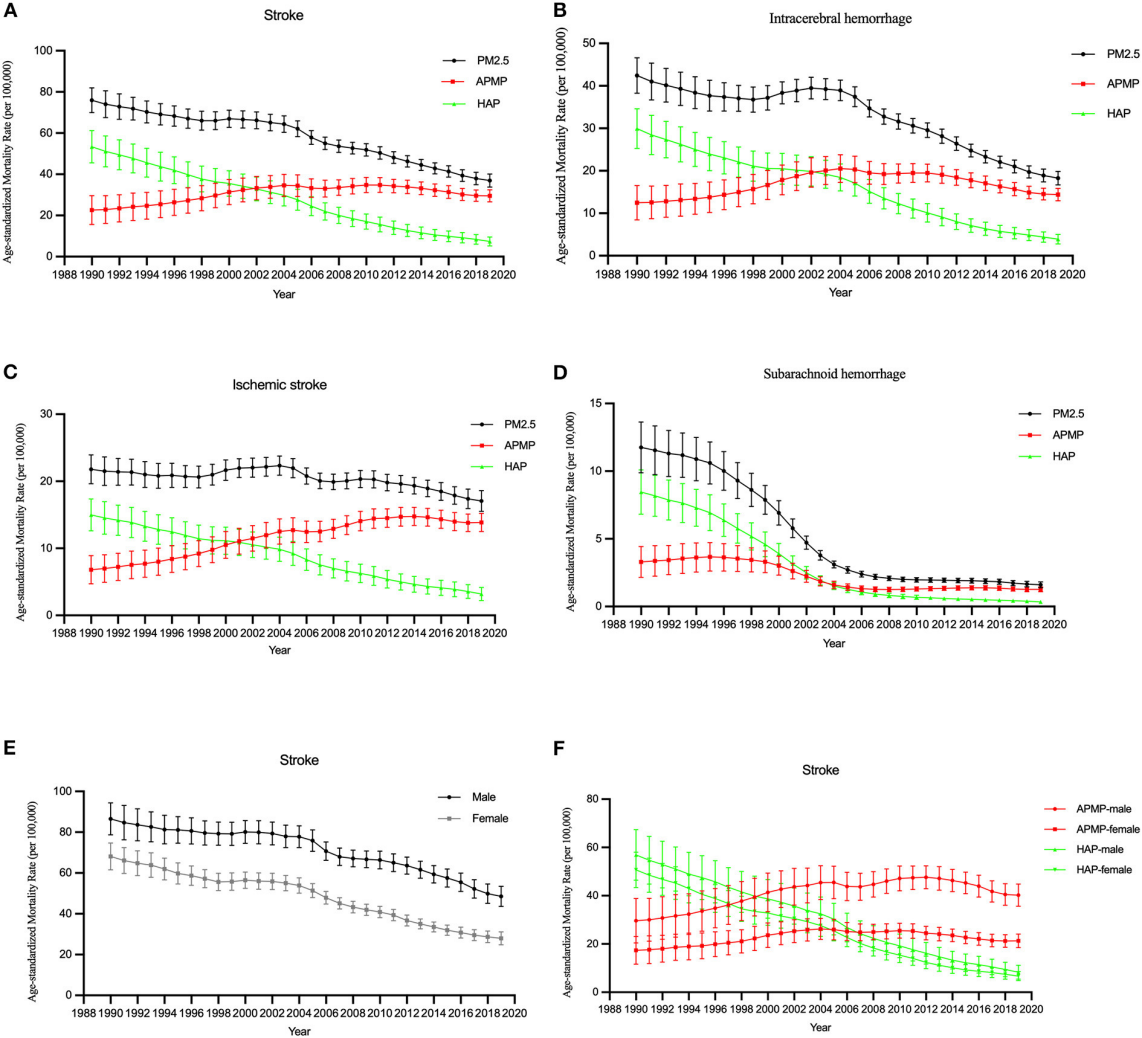
The temporal trends in the ASMR of stroke attributable to PM2.5 exposure from 1990 to 2019. **(A–D)** The mortality rate of stroke and subtypes due to PM2.5 exposure, ambient particulate matter pollution, and household air pollution for both sexes. **(E)** The mortality rate of stroke due to PM2.5 exposure by sex. **(F)** The mortality rate of stroke due to ambient particulate matter pollution (APMP) and household air pollution (HAP) by sexes.

The ASMR due to ambient particulate matter pollution was higher than that due to household air pollution after 2002. For males, the ASMR due to ambient particulate matter pollution was higher than that due to household air pollution after 1999, and for females, the ASMR due to ambient particulate matter pollution was higher than that due to household air pollution after 2005 (Table 1; Figure 1).

**Table 1:** The results by Joinpoint regression analysis on the mortality rate of stroke, intracerebral hemorrhage, ischemic stroke and subarachnoid hemorrhage attributable to PM2.5 exposure from 1990 to 2019.

		**PM2.5**		**Ambient particulate matter pollution**		**Household air pollution**	
		**AAPC (%)**	**95%CI**	**AAPC (%)**	**95%CI**	**AAPC (%)**	**95%CI**
Stroke	Both sexes	−2.5*	(-2.6,−2.3)	1.0*	(0.6,1.5)	−6.6*	(-6.9,−6.3)
	Male	−2.0*	(-2.2,−1.7)	1.1*	(0.6,1.6)	−6.4*	(-6.8,−6.1)
	Female	−3.0*	(-3.3,−2.8)	0.7*	(0.4,1.0)	−6.7*	(-7.1,−6.2)
Intracerebral hemorrhage	Both sexes	−2.8*	(-3.2,−2.5)	0.5*	(0.3,0.7)	−6.7*	(-7.2,−6.3)
	Male	−2.4*	(-2.7,−2.2)	0.6*	(0.1,1.0)	−6.5*	(-6.7,−6.4)
	Female	−3.4*	(-3.7,−3.2)	0.3	(-0.0,0.6)	−6.9*	(-7.3,−6.6)
Ischemic stroke	Both sexes	−0.8*	(-1.1,−0.6)	2.5*	(2.0,2.9)	−5.1*	(-5.5,−4.8)
	Male	−0.5*	(-0.7,−0.2)	2.5*	(2.1,2.9)	−5.2*	(-5.6,−4.8)
	Female	−1.3*	(-1.5,−1.1)	2.4*	(1.8,3.0)	−5.3*	(-5.6,−4.9)
Subarachnoid hemorrhage	Both sexes	−6.7*	(-7.0,−6.3)	−3.3*	(-3.7,−2.8)	−10.5*	(-10.8,−10.1)
	Male	−6.1*	(-6.4,−5.7)	−3.1*	(-3.5,−2.6)	−10.1*	(-10.4,−9.9)
	Female	−7.2*	(-7.6,−6.8)	−3.7*	(-4.1,−3.2)	−10.6*	(-11.0,−10.3)

^*^Statistically significant (p<*0.05); AAPC, average annual percent change*.

### The age-period-cohort analysis of the mortality rate of stroke attributable to PM2.5 exposure

**Figure 2:**
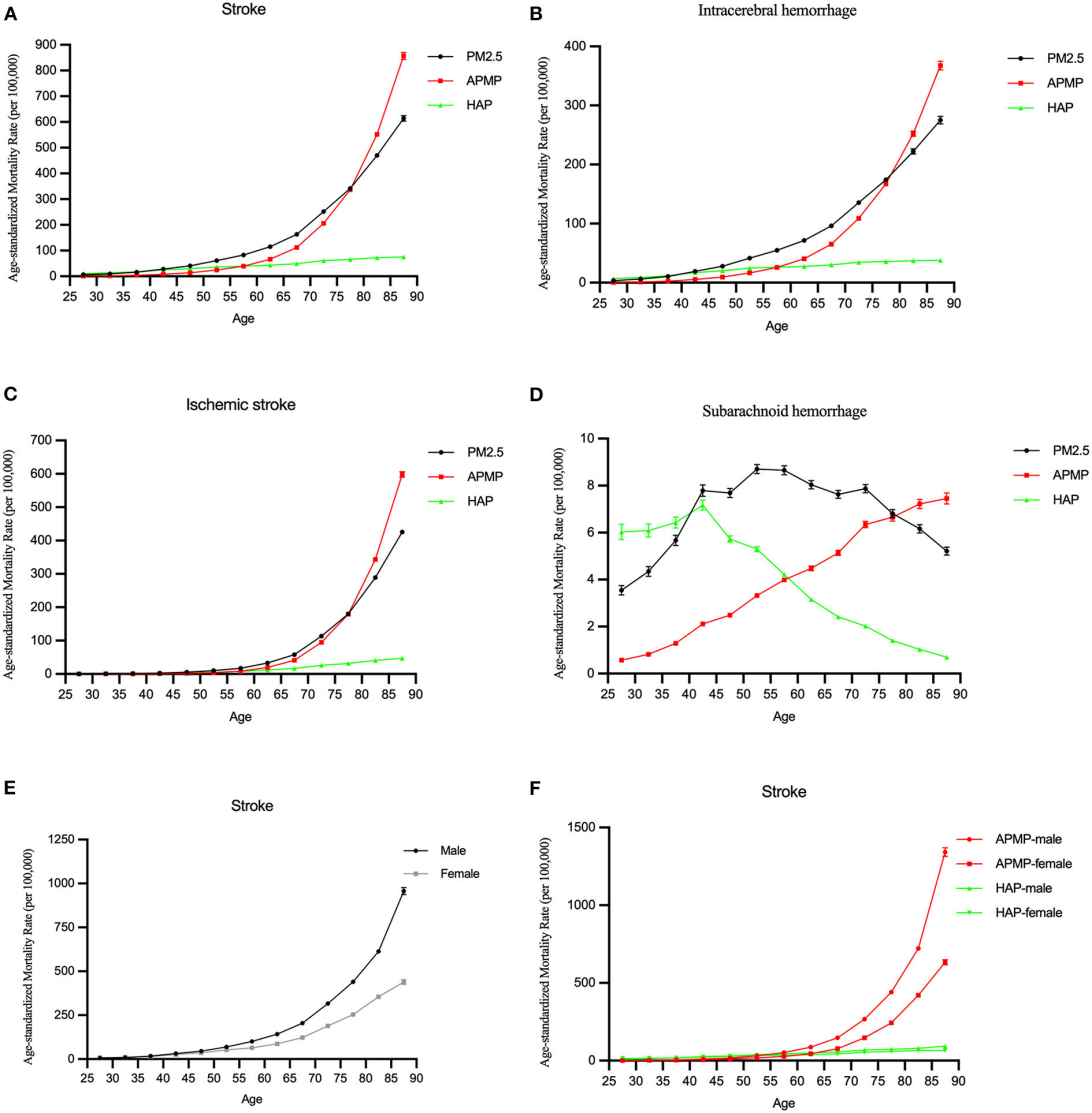
The longitudinal age curves of the mortality rate of stroke attributable to PM2.5 exposure from 1990 to 2019. **(A–D)** The longitudinal age curves of stroke and subtypes for both sexes in PM2.5 exposure. **(E)** The longitudinal age curves of stroke by sex in PM2.5 exposure. **(F)** The longitudinal age curves of stroke by sex in ambient particulate matter pollution (APMP) and household air pollution (HAP).

For the same birth cohort, the mortality rate of stroke attributable to PM2.5 increased with age. The mortality rate of stroke among males was higher than that among females (Figures 2A,E).

Among different subtypes, for subarachnoid hemorrhage, the mortality rate was lower than others, and it increased with age before 55 and slightly decreased after that. Other subtypes of stroke had increasing trends with age attributable to PM2.5, they trended upwards rapidly after the 55–60 age groups (Figures 2B–D).

In terms of ambient particulate matter pollution and household air pollution, the mortality rates due to ambient particulate matter pollution and household air pollution were low in younger group, but the mortality rate due to ambient particulate matter pollution significantly increased with age among those older groups. Similar changes were observed in males and females (Figures 2A,F).

**Figure 3:**
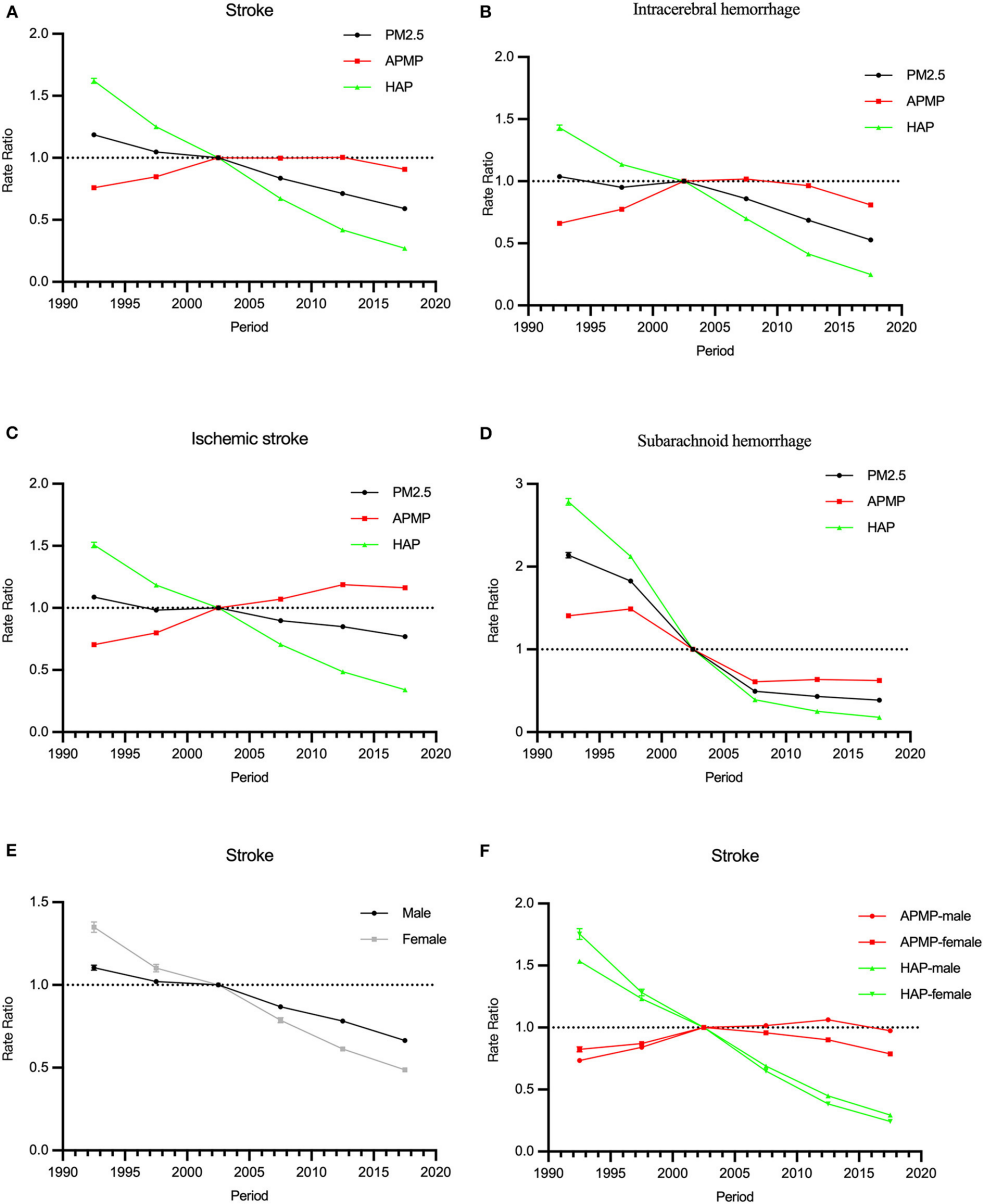
The period RRs of the mortality rate of stroke attributable to PM2.5 exposure from 1990 to 2019. **(A–D)** The period RRs of stroke and subtypes for both sexes in PM2.5 exposure. **(E)** The period RRs of stroke by sex in PM2.5 exposure. **(F)** The period RRs of stroke by sex in ambient particulate matter pollution (APMP) and household air pollution (HAP).

The period RRs of all stroke subtypes attributable to PM2.5 exposure trended downwards. The downward trend among females was steeper than that among males (Figures 3A,E,F).

In terms of ambient particulate matter pollution, the period RRs of stroke increased from 1990 to 2019. For household air pollution, it trended downwards significantly. Similar trends were observed among those of intracerebral hemorrhage and ischemic stroke. The period RRs of subarachnoid hemorrhage attributable to ambient particulate matter pollution and household air pollution decreased from 1990 to 2019 (Figures 3B–D).

**Figure 4:**
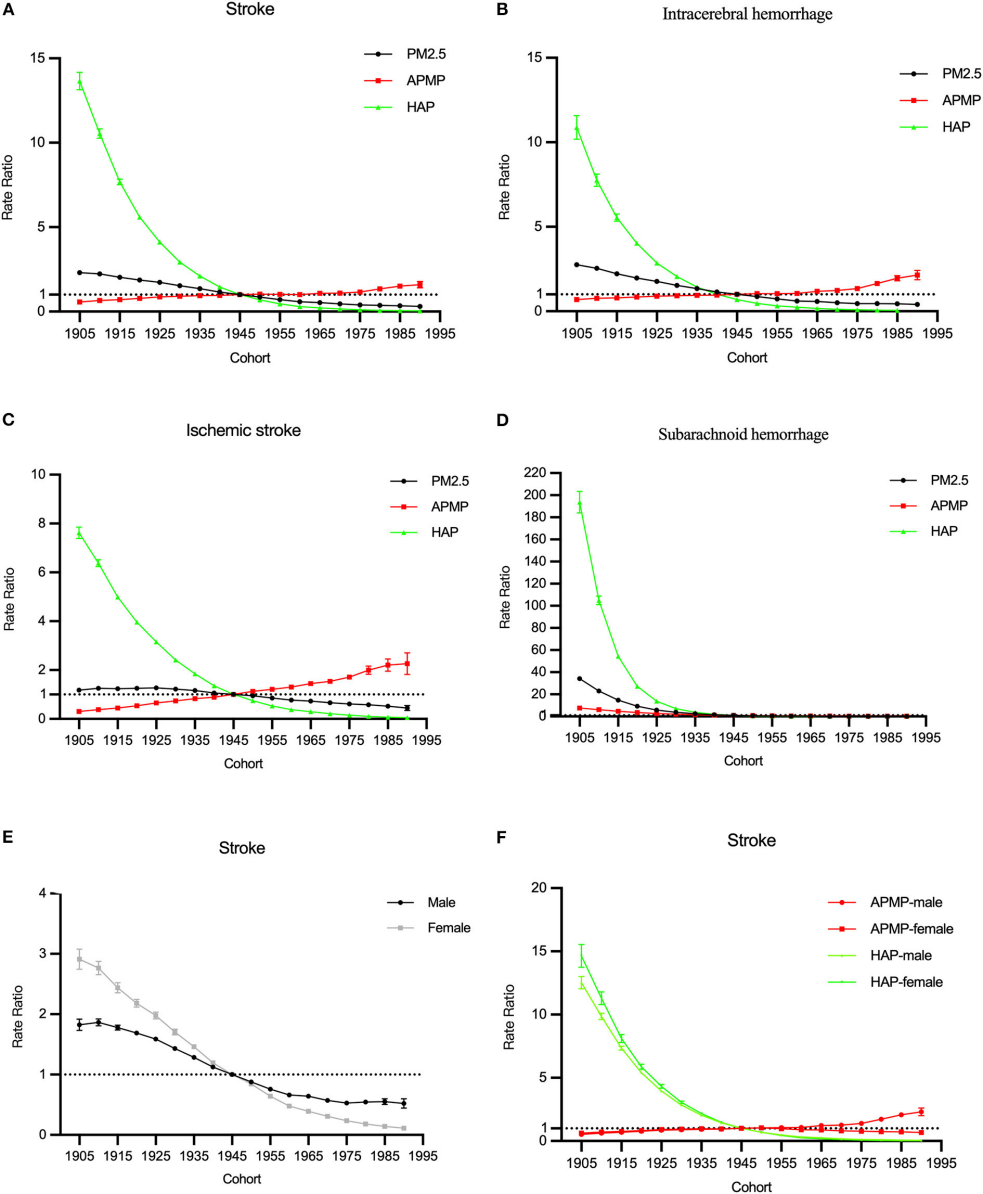
The cohort RRs of the mortality rate of stroke attributable to PM2.5 exposure from 1990 to 2019. **(A–D)** The cohort RRs of stroke and subtypes for both sexes in PM2.5 exposure. **(E)** The cohort RRs of stroke by sex in PM2.5 exposure. **(F)** The cohort RRs of stroke by sex in ambient particulate matter pollution (APMP) and household air pollution (HAP).

Cohort RRs of all stroke subtypes attributable to PM2.5 exposure decreased among those born from 1905 to 1990. The cohort effects were opposites for ambient particulate matter pollution and household air pollution: for ambient particulate matter pollution, the cohort effects increased, for household air pollution, they declined. Similar trends were observed among both males and females. The downward trend among females was steeper than that among males (Figures 4A,E,F).

In terms of different subtypes, the cohort RRs for household air pollution trended downwards obviously, while for ambient particulate matter pollution, the cohort RRs of the mortality rate of intracerebral hemorrhage and ischemic stroke trended upwards (Figures 4B–D).

The overall net drift values of all stroke subtypes attributable to PM2.5 were below 0 (*P* < 0.001). However, the net drift values of intracerebral hemorrhage and ischemic stroke attributable to ambient particulate matter pollution were above 0 (0.96 and 2.17%, respectively, *P* < 0.001). In terms of sexes, the net drift value of stroke for males was−1.97%, and for females, it was−3.98% (*P* < 0.001). Among different stroke subtypes, males had higher net drift values, compared with females.

**Figure 5:**
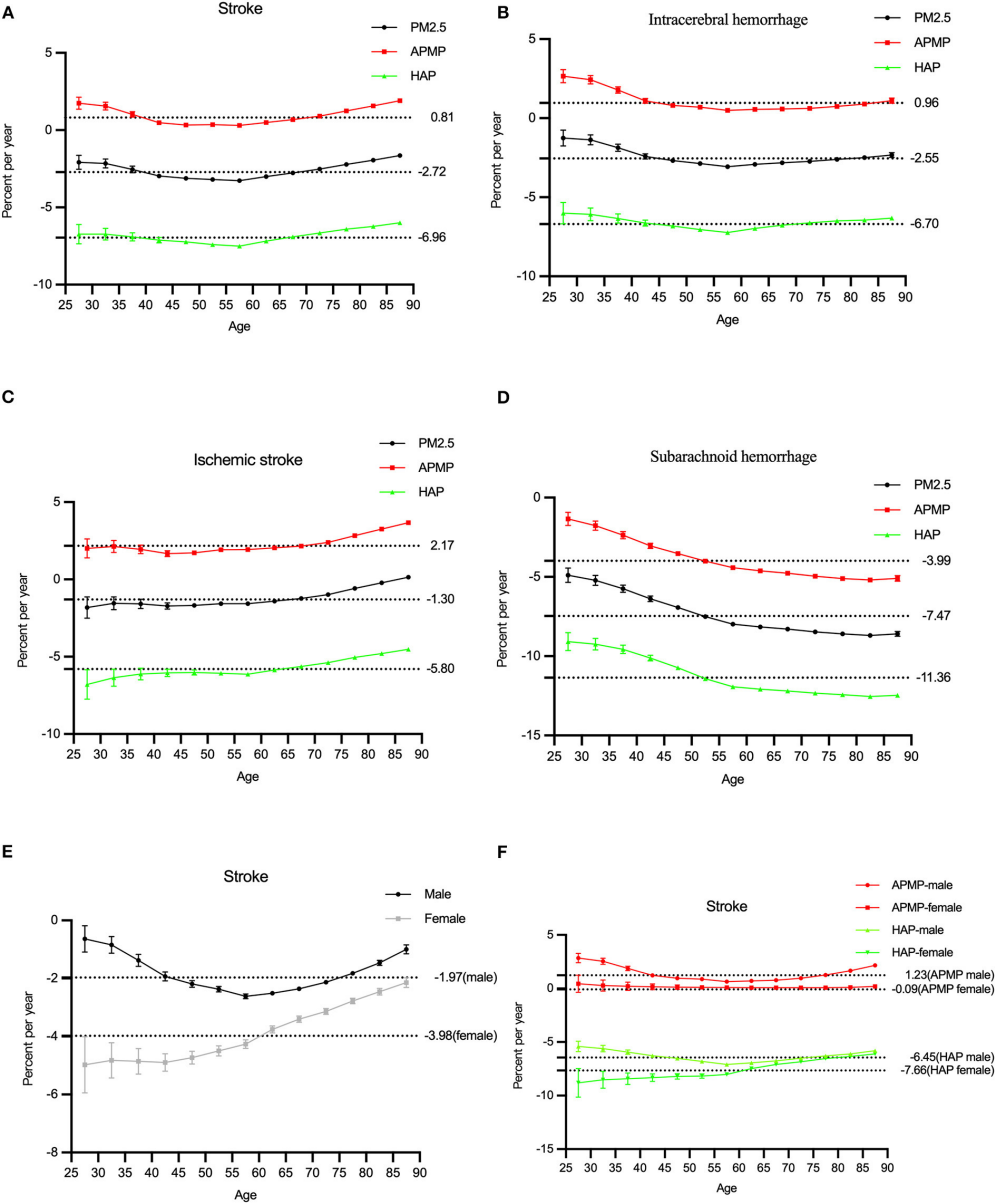
The local drifts of the mortality rate of stroke attributable to PM2.5 exposure from 1990 to 2019. **(A–D)** The local drifts of stroke and subtypes for both sexes in PM2.5 exposure. **(E)** The local drifts of stroke by sex in PM2.5 exposure. **(F)** The local drifts of stroke by sex in ambient particulate matter pollution (APMP) and household air pollution (HAP).

The local drift values of stroke decreased by age before the 60-age group and increased after that. Similar changes were observed for ambient particulate matter pollution and household air pollution, while the local drift values of intracerebral hemorrhage decreased by age, and for ischemic stroke, it increased by age (Figure 5).

The detailed results of the APC model regarding the age, period, and cohort effect were shown in Supplementary Tables 1–5.

## Discussion

This study provided long-term estimates of the mortality rate of stroke attributable to PM2.5 exposure by sex, age, and stroke subtypes, clarifying the impact of ambient particulate matter pollution and household air pollution changes on stroke by Joinpoint regression and age-period-cohort model in China.

We found that, over 30 years, the number of stroke cases attributable to PM2.5 substantially increased, and the number of deaths attributable to ambient particulate matter pollution accounted for most of it. According to the mortality rate, there is a reduction in the ASMRs of stroke due to PM2.5 from 1990 to 2019, but it also shows the increase in the ASMRs due to ambient particulate matter pollution which was significantly higher than those due to household air pollution after 2002. This indicates that ambient particulate matter pollution has replaced household air pollution as the main risk factor of PM2.5 related to stroke. Further reduction in anthropogenic emissions is needed to accelerate the decrease in ambient PM2.5 concentrations, or the burden of stroke attributable to PM2.5 is still severe. Moreover, many rural residents, particularly in western, central, and north-eastern China, still rely heavily on solid fuels, causing uneven household air pollution in the population of China, which should be also called attention to Ma et al. (17).

Both sexes had similar trends in the ASMRs attributable to ambient particulate matter pollution and household air pollution from 1990 to 2019, but the mortality rate of stroke attributable to PM2.5 was higher among males than females. The ASMR attributable to ambient particulate matter pollution among males reached its highest in 2012, and it slightly reduced after that. These findings suggest that PM2.5 exposure had a greater adverse function on the mortality rate of stroke among males, but this effect has been moderated by improvements for both indoor and outdoor pollution in recent years. Another notable aspect is that air pollution is also associated with smoking (38), contributing to the heavier disease burden among males than females.

In subgroup analyses for stroke subtypes, we found that the ASMRs trends of all stroke subtypes attributable to PM2.5 were decreased, but for ambient particulate matter pollution, the ASMRs of ischemic stroke substantially increased. This may be because exposure to ambient particulate matter pollution may induce acceleration of atherosclerosis, alter the vasomotor tone, cause vascular inflammation, and promote blood coagulation, which pathophysiological changes are more likely to provoke ischemic stroke, compared with hemorrhagic stroke (39–41).

We further analyzed the effects of age, period, and cohort on the epidemiological changes in stroke attributable to PM2.5 exposure. We found that the ASMRs of ischemic stroke and intracerebral hemorrhage due to both ambient particulate matter pollution and household air pollution significantly increased after their levels among those 55–60 years, and were low among the younger age groups. A study also estimated that the health cost of stroke attributable to PM2.5 increased faster in the older population than in the younger population (7). Therefore, more policy actions are needed to reduce the stroke burden attributable to PM2.5 concentrations to which elderly individuals are exposed. In addition, the mortality of subarachnoid hemorrhage increased with age before 55 and slightly decreased after that. This finding might be because the ratio of hemorrhagic stroke cases is higher among younger populations overall stroke cases, and hemorrhagic stroke is more preventable (42).

The period RRs attributable to PM2.5 trended downward among all subtypes, but there was still an obvious upward trend for ischemic stroke due to ambient particulate matter pollution, indicating that despite the improvement of air pollution in China, government should formulate an effective policy to prevent the adverse result of ischemic stroke due to ambient particulate matter pollution. The cohort RRs attributable to PM2.5 decreased among those born from 1905 to 1990. But for ambient particulate matter pollution, the cohort RRs trend increased among those born from 1905 to 1990, indicating the younger generations may have a higher risk to get stroke due to ambient particulate matter pollution and we should pay attention to the long-term damage of it.

The overall net values of all stroke subtypes attributable to PM2.5 exposure was−2.72%, but the net drift value of ischemic stroke attributable to ambient particulate matter pollution was 2.17%. Considering all results in the local drift value, formulate effective policies for outdoor air pollution attributable to ischemic stroke, males and the elderly might remarkably reduce the risk of stroke from air pollution in the future.

Despite some advancements in this study, there are several limitations. First, although GBD 2019 has modified and adjusted its data sources and collection and evaluation method to improve data quality, it could be difficult to avoid the bias caused by missing data. Second, studies on the relationship between PM2.5 and different types of stroke might be lacking, which led to some mixed results explaining the difference among stroke subtypes (10, 39, 40, 43, 44). Therefore, more studies are needed to improve the reliability of the results through method standardization, increasing sample size, and stratified analysis. Third, in addition to the effects of age, period, cohort, and gender, we should take more factors into account, such as regional differences between North and South in China (45), economic development, and education level.

## Conclusion

In summary, ambient air pollution has become the main type of PM2.5 leading to stroke, and its exposure is more harmful to ischemic stroke, males, and elderly in China. Young generation may have a higher risk to get stroke in the future. To reduce the burden of stroke attributable to PM2.5, Chinese government should pay attention to the impact of outdoor air pollution on stroke and take effective public health policies and interventions to protect the specific population.

## Data availability statement

The original contributions presented in the study are included in the article/Supplementary material, further inquiries can be directed to the corresponding author.

## Author contributions

HC and ZL wrote the manuscript, with contributions from SZ and GZ. ZZ and SL conducted the data collection. HC and JZ did the analysis. All authors contributed to data interpretation, wrote and revised various parts of the article, and approved the submitted version.

## Funding

This study was funded by the National Natural Science Foundation of China (Grant Numbers: 81973979, 71774049, 71273083, and 71333005), Natural Science Foundation of Guangdong Province (Grant Number: 2019A1515011496), Social Science Foundation of Guangdong Province (Grant Number: GD19CSH04), and the Key Projects of Philosophy and Social Sciences Research of Education Department of Hubei in China (Grant Number: 17ZD024).

## Conflict of interest

The authors declare that the research was conducted in the absence of any commercial or financial relationships that could be construed as a potential conflict of interest.

## Publisher's note

All claims expressed in this article are solely those of the authors and do not necessarily represent those of their affiliated organizations, or those of the publisher, the editors and the reviewers. Any product that may be evaluated in this article, or claim that may be made by its manufacturer, is not guaranteed or endorsed by the publisher.

## References

[1] Kim J Thayabaranathan T Donnan GA Howard G Howard VJ Rothwell PM. Global stroke statistics 2019. Int J Stroke. (2020) 15:819–38. 10.1177/1747493020909545 32146867

[2] Ma Q Li R Wang L Yin P Wang Y Yan C. Temporal trend and attributable risk factors of stroke burden in China, 1990–2019: an analysis for the global burden of disease study 2019. Lancet Public Health. (2021) 6:e897–906. 10.1016/S2468-2667(21)00228-0 34838196 PMC9047702

[3] Danaei G Finucane MM Lu Y Singh GM Cowan MJ Paciorek CJ. National, regional, and global trends in fasting plasma glucose and diabetes prevalence since 1980: systematic analysis of health examination surveys and epidemiological studies with 370 country-years and 2·7 million participants. Lancet. (2011) 378:31–40. 10.1016/S0140-6736(11)60679-X 21705069

[4] Feigin VL Roth GA Naghavi M Parmar P Krishnamurthi R Chugh S. Global burden of stroke and risk factors in 188 countries, during 1990-2013: a systematic analysis for the global burden of disease study 2013. Lancet Neurol. (2016) 15:913–24. 10.1016/S1474-4422(16)30073-4 27291521

[5] Collaborators GRF. Global burden of 87 risk factors in 204 countries and territories, 1990–2019: a systematic analysis for the global burden of disease study 2019. Lancet. (2020) 396:1223–49. 10.1016/S0140-6736(20)30752-2 33069327 PMC7566194

[6] Di Q Wang Y Zanobetti A Wang Y Koutrakis P Choirat C. Air pollution and mortality in the medicare population. N Engl J Med. (2017) 376:2513–22. 10.1056/NEJMoa1702747 28657878 PMC5766848

[7] Yin H Brauer M Zhang JJ Cai W Navrud S Burnett R. Population ageing and deaths attributable to ambient Pm(2·5) pollution: a global analysis of economic cost. Lancet Planet Health. (2021) 5:e356–e67. 10.1016/S2542-5196(21)00131-5 34119010

[8] Collaborators GRF. Global, regional, and national comparative risk assessment of 84 behavioural, environmental and occupational, and metabolic risks or clusters of risks for 195 countries and territories, 1990-2017: a systematic analysis for the global burden of disease study 2017. Lancet. (2018) 392:1923–94. 10.1016/S0140-6736(18)32225-6 30496105 PMC6227755

[9] Stafoggia M Cesaroni G Peters A Andersen ZJ Badaloni C Beelen R. Long-term exposure to ambient air pollution and incidence of cerebrovascular events: results from 11 European cohorts within the escape project. Environ Health Perspect. (2014) 122:919–25. 10.1289/ehp.1307301 24835336 PMC4153743

[10] Huang K Liang F Yang X Liu F Li J Xiao Q. Long term exposure to ambient fine particulate matter and incidence of stroke: prospective cohort study from the China-par project. BMJ. (2019) 367:l6720. 10.1136/bmj.l6720 31888885 PMC7190010

[11] Liu Z Hystad P Zhang Y Rangarajan S Yin L Wang Y. Associations of household solid fuel for heating and cooking with hypertension in Chinese adults. J Hypertens. (2021) 39:667–76. 10.1097/HJH.0000000000002689 33186328

[12] Yu K Qiu G Chan KH Lam KH Kurmi OP Bennett DA. Association of solid fuel use with risk of cardiovascular and all-cause mortality in rural China. JAMA. (2018) 319:1351–61. 10.1001/jama.2018.2151 29614179 PMC5933384

[13] Aunan K Hansen MH Wang S. Introduction: air pollution in China. China Q. (2017) 234:279–98. 10.1017/S0305741017001369

[14] Yang X Zhang T Zhang X Chu C Sang S. Global burden of lung cancer attributable to ambient fine particulate matter pollution in 204 countries and territories, 1990-2019. Environ Res. (2022) 204:112023. 10.1016/j.envres.2021.112023 34520750

[15] Li X Hussain SA Sobri S Md Said MS. Overviewing the air quality models on air pollution in sichuan basin, China. Chemosphere. (2021) 271:129502. 10.1016/j.chemosphere.2020.129502 33465622

[16] Luo L Jiang J Zhang G Wang L Wang Z Yang J. Stroke mortality attributable to ambient particulate matter pollution from 1990 to 2015 in China: an age-period-cohort and spatial autocorrelation analysis. Int J Environ Res Public Health. (2017) 14:772. 10.3390/ijerph14070772 28703768 PMC5551210

[17] Ma Y Yang D Bai J Zhao Y Hu Q Yu C. Time trends in stroke and subtypes mortality attributable to household air pollution in Chinese and Indian adults: an age-period-cohort analysis using the global burden of disease study 2019. Front Aging Neurosci. (2022) 14:740549. 10.3389/fnagi.2022.740549 35250534 PMC8895296

[18] Ban J Wang Q Ma R Zhang Y Shi W Zhang Y. Associations between Short-term exposure to Pm2.5 and stroke incidence and mortality in China: a case-crossover study and estimation of the burden. Environ Pollut. (2021) 268:115743. 10.1016/j.envpol.2020.115743 33022547

[19] Yang X Zhang L Chen X Liu F Shan A Liang F. Long-term exposure to ambient Pm25 and stroke mortality among urban residents in Northern China. Ecotoxicol Environ Saf. (2021) 213:112063. 10.1016/j.ecoenv.2021.112063 33636465 PMC8150861

[20] Zhou M Wang H Zhu J Chen W Wang L Liu S. Cause-specific mortality for 240 causes in China during 1990-2013: a systematic subnational analysis for the global burden of disease study 2013. Lancet. (2016) 387:251–72. 10.1016/S0140-6736(15)00551-6 26510778

[21] Network GBoDC. Global Burden of Disease Study 2019 (Gbd 2019) Cause List Mapped to Icd Codes. Institute for Health Metrics and Evaluation (IHME) (2020).

[22] Collaborators GMaCoD. Global, regional, and national life expectancy, all-cause mortality, and cause-specific mortality for 249 causes of death, 1980-2015: a systematic analysis for the global burden of disease study 2015. Lancet. (2016) 388:1459–544. 10.1016/S0140-6736(16)31012-1 27733281 PMC5388903

[23] Burnett RT Pope CA 3rd Ezzati M Olives C Lim SS Mehta S. An integrated risk function for estimating the global burden of disease attributable to ambient fine particulate matter exposure. Environ Health Perspect. (2014) 122:397–403. 10.1289/ehp.1307049 24518036 PMC3984213

[24] Collaborators GCoD. Global, regional, and national age-sex-specific mortality for 282 causes of death in 195 countries and territories, 1980-2017: a systematic analysis for the global burden of disease study 2017. Lancet. (2018) 392:1736–88. 10.1016/S0140-6736(18)32203-7 30496103 PMC6227606

[25] Rudd KE Johnson SC Agesa KM Shackelford KA Tsoi D Kievlan DR. Global, regional, and national sepsis incidence and mortality, 1990-2017: analysis for the global burden of disease study. Lancet. (2020) 395:200–11. 10.1016/S0140-6736(19)32989-7 31954465 PMC6970225

[26] Li HZ Du LB. Application of Joinpoint regression model in cancer epidemiological time trend analysis. Chin J Prev Med. (2020) 54:908–12. 10.3760/cma.j.cn112150-20200616-00889 32842323

[27] Robertson C Gandini S Boyle P. Age-period-cohort models: a comparative study of available methodologies. J Clin Epidemiol. (1999) 52:569–83. 10.1016/S0895-4356(99)00033-5 10408997

[28] Chen YT Wu CY Li YL Chen LY Chiou HY. Time trends in psoriasis and psoriatic arthritis incidence from 2002 to 2016 in Taiwan: an age-period-cohort analysis. J Clin Med. (2022) 11:3744. 10.3390/jcm11133744 35807026 PMC9267639

[29] Martínez-Alés G Gimbrone C Rutherford C Keyes K López-Cuadrado T. Role of foreign-born status on suicide mortality in spain between 2000 and 2019: an age-period-cohort analysis. Int J Public Health. (2022) 67:1604538. 10.3389/ijph.2022.1604538 35664647 PMC9156625

[30] Yang Y Schulhofer-Wohl S Fu WJ Land KC. The intrinsic estimator for age-period-cohort analysis: what it is and how to use it. Am J Sociol. (2008) 113:1697–1736. 10.1086/587154

[31] Wu X Zhu B Zhou J Bi Y Xu S Zhou B. The epidemiological trends in the burden of lung cancer attributable to Pm25 exposure in China. BMC Public Health. (2021) 21:737. 10.1186/s12889-021-10765-1 33858412 PMC8051098

[32] Yang J Zhang YQ Luo LS Meng RT Yu CH. Global mortality burden of cirrhosis and liver cancer attributable to injection drug use, 1990-2016: an age-period-cohort and spatial autocorrelation analysis. Int J Environ Res Public Health. (2018) 15:170. 10.3390/ijerph15010170 29361804 PMC5800269

[33] Cui Y Mubarik S Li R Nawsherwan Yu C. Trend dynamics of thyroid cancer incidence among China and the U.S. adult population from 1990 to 2017: a joinpoint and age-period-cohort analysis. BMC Public Health. (2021) 21:624. 10.1186/s12889-021-10635-w 33789605 PMC8010947

[34] Dhamnetiya D Patel P Jha RP Shri N Singh M Bhattacharyya K. Trends in incidence and mortality of tuberculosis in India over past three decades: a joinpoint and age-period-cohort analysis. BMC Pulm Med. (2021) 21:375. 10.1186/s12890-021-01740-y 34784911 PMC8597252

[35] Luo LY. Assessing validity and application scope of the intrinsic estimator approach to the age-period-cohort problem. Demography. (2013) 50:1945–67. 10.1007/s13524-013-0243-z 24072610 PMC5129181

[36] Liu XX Jiang JF Yu CH Wang YB Sun Y Tang J. Secular trends in incidence and mortality of bladder cancer in China, 1990-2017: a joinpoint and age-period-cohort analysis. Cancer Epidemiol. (2019) 61:95–103. 10.1016/j.canep.2019.05.011 31176961

[37] Rosenberg PS Check DP Anderson WF. A Web tool for age-period-cohort analysis of cancer incidence and mortality rates. Cancer Epidemiol Biomarkers Prev. (2014) 23:2296–302. 10.1158/1055-9965.EPI-14-0300 25146089 PMC4221491

[38] Collaborators IS-LDBIAP. The impact of air pollution on deaths, disease burden, and life expectancy across the states of India: the global burden of disease study 2017. Lancet Planet Health. (2019) 3:e26–39. 10.1016/S2542-5196(18)30261-4 30528905 PMC6358127

[39] Tian Y Liu H Wu Y Si Y Song J Cao Y. Association between ambient fine particulate pollution and hospital admissions for cause specific cardiovascular disease: time series study in 184 major Chinese cities. BMJ. (2019) 367:l6572. 10.1136/bmj.l6572 31888884 PMC7190041

[40] Qiu H Sun S Tsang H Wong CM Lee RS Schooling CM. Fine particulate matter exposure and incidence of stroke: a cohort study in Hong Kong. Neurology. (2017) 88:1709–17. 10.1212/WNL.0000000000003903 28363975

[41] Hajat A Allison M Diez-Roux AV Jenny NS Jorgensen NW Szpiro AA. Long-term exposure to air pollution and markers of inflammation, coagulation, and endothelial activation: a repeat-measures analysis in the multi-ethnic study of atherosclerosis (mesa). Epidemiology. (2015) 26:310–20. 10.1097/EDE.0000000000000267 25710246 PMC4455899

[42] Wang Y Zhou L Guo J Wang Y Yang Y Peng Q. Secular trends of stroke incidence and mortality in China, 1990 to 2016: the global burden of disease study 2016. J Stroke Cerebrovasc Dis. (2020) 29:104959. 10.1016/j.jstrokecerebrovasdis.2020.104959 32689583

[43] Noh J Sohn J Han M Kang DR Choi YJ Kim HC. Long-term effects of cumulative average Pm25 exposure on the risk of hemorrhagic stroke. Epidemiology. (2019) 30(Suppl 1):S90–8. 10.1097/EDE.0000000000001001 31181011

[44] Li J Zhang X Yin P Wang L Zhou M. Ambient fine particulate matter pollution and years of life lost from cardiovascular diseases in 48 large Chinese cities: association, effect modification, and additional life gain. Sci Total Environ. (2020) 735:139413. 10.1016/j.scitotenv.2020.139413 32480149

[45] Liu H Tian Y Xu Y Zhang J. Ambient particulate matter concentrations and hospitalization for stroke in 26 Chinese cities: a case-crossover study. Stroke. (2017) 48:2052–9. 10.1161/STROKEAHA.116.016482 28663508

